# SHP Buddy: a QGIS plugin for generating shapefiles to support remote sensing in plant breeding and agronomic experiments

**DOI:** 10.1186/s13007-025-01336-1

**Published:** 2025-02-12

**Authors:** Nathaniel Burner, Donna K. Harris, Zenglu Li

**Affiliations:** 1 Institute of Plant Breeding, Genetics and Genomics, Department of Crop and Soil Sciences, University of Georgia, Athens, GA USA; 2 Department of Plant Sciences, Sheridan Research and Extension Center, University of Wyoming, Sheridan, WY USA

**Keywords:** Shapefiles, High-throughput phenotyping, Remote sensing, Unmanned aerial vehicles (UAVs), Field layout

## Abstract

**Background:**

Shapefiles are a geospatial vector data format used to indicate geographic features in geographic information systems (GIS) software. Shapefiles are used in high-throughput phenotyping plant breeding and agronomic studies to identify plots from aerial imagery and extract remote sensing data. However, the process of manually creating shapefiles is tedious and error prone. Current options that assist in shapefile generation suffer from issues such as installation processes that require a degree of programming knowledge or inefficient methods for incorporating plot-level information from field books. In this study, we have developed a program called ‘SHP Buddy’, a QGIS plugin that provides accessible and intuitive functions that quickly generate shapefiles for common experimental layouts used in agricultural research.

**Results:**

SHP Buddy is a free and open source QGIS plugin that is easily downloaded directly from the QGIS plugin repository. It provides options for generating serpentine replicated and unreplicated experimental layouts. Further, SHP Buddy is the first of its type to provide an intuitive method for removing non-experimental plots, such as non-experimental “fill” plots at the end of experiments or plots in irrigation wheel tracks. Plot information is easily incorporated by uploading a field book CSV file that contains a column of matching plot numbers. Lastly, plot dimensions can be modified to produce more precise regions of interest.

**Conclusions:**

SHP Buddy substantially reduces the time and increases the accuracy of shapefile generation. This results in reliable shapefiles that improve record keeping and the quality of high-throughput phenotyping data extracted. By working natively in QGIS, SHP Buddy provides an efficient solution to shapefile generation while maintaining a low learning curve.

## Background

The use of unmanned aerial vehicles (UAVs) for remote sensing in plant science research and agricultural production has proliferated over the past decade because of their ability to image large areas of crop fields in minutes [1–2]. UAVs are a popular technology in precision agriculture that enable farmers to monitor variation in agronomic traits within fields to aid in management decisions regarding the timing, rate, and location of irrigation and pesticide and fertilizer applications, in addition to prescribing management practices to improve farmland quality in subsequent seasons [3]. Precision agriculture offers numerous economic and environmental benefits to farmers by optimizing inputs and cultural practices to areas that will most benefit crop health, yield, and other traits of interest [4]. Additionally, farmers can use aerial imagery to identify areas with issues such as low seedling emergence, lodging, disease, pest, weed pressure, nutrient deficiencies, and flooding [5]. This is particularly useful when the whole or part of a field is inaccessible to humans.

Remote sensing has been steadily adopted by plant breeders and agronomists to reduce the time, labor, and error associated with traditional phenotyping [6]. Breeding programs need precise phenotyping methods for identifying and advancing superior performing genotypes. In most crops, performance is measured by yield in replicated experiments across multiple locations and years. However, yield is a low heritability trait that is influenced by the expression and interaction of thousands of other genes expressed over the course of a plant’s life cycle in addition to environmental and management factors [7]. The individual phenotypic effects of some of these genes may be highly heritable, however phenotyping for minor traits is challenging due to the need for destructive phenotyping, lack of accurate phenotyping methods or equipment, time and resource constraints, or lack of visibility [6]. Remote sensing is an attractive option for breeders as it allows for the non-destructive capturing of spectral information that can be associated with many different physiological and morphological traits [8]. This provides breeders with more objective phenotypic information from which to base selections. More effective selection methods increase the efficiency of developing better performing cultivars for farmers [9].

Plant breeding programs require large population sizes to increase the probability of identifying desirable genotypes [10]. Therefore, it is common for thousands of unique genotypes to be evaluated across replicated trials. Deriving spectral phenotypes from aerial imagery requires accurate demarcation of test plots in these experiments [1–2]. Geospatial vectors, known as shapefiles, of plot boundaries can be manually generated with most geographic information system (GIS) programs such as QGIS and ArcGIS [11–13]. A typical shapefile for a plant breeding experiment will consist of a grid of rectangles corresponding to individual plots.

Manually generating shapefiles for fields with a large number of experiments and plots is a tedious and time-consuming process that is prone to error [14]. For example, a plant breeding program will typically consist of multiple yield trials in the later stages of the breeding pipeline by grouping varieties with similar characteristics (such as maturity) and breeding objectives. A typical workflow for creating plant breeding plot shapefiles begins with creating a ‘fishnet’ grid. In GIS terminology, a fishnet is an area of aerial imagery that is subdivided into rectangular cells. In the context of agricultural and plant breeding experiments, the extent of the fishnet is the experiment, and the cells are individual plots. Incorporating plot information such as line name, pedigree information, and other phenotypic information requires sorting plots by position then matching values in a spreadsheet software outside of the program then reimported to the GIS software. This process is complicated by the lack of a convenient way to arrange raw cell positions in a serpentine pattern, which is typically how plots are ordered. Lastly, further manual scaling of plot dimensions may be necessary to better exclude unwanted regions such as adjacent plots and alleyways from the region of interest.

Currently, there is a lack of convenient and intuitive options for quickly generating shapefiles for common plant breeding experiments and importing plot information. The few options currently available present inconveniences to the user experience such as installations that require a degree of programming knowledge, requiring field map files to incorporate field book information, or lack of an automated way to remove non-experimental “fill” plots [15–18]. In agricultural experiments such as plant breeding yield trials, fill plots are planted with a generic crop variety in lieu of leaving a plot barren and are therefore is generally not phenotyped. This situation occurs when the number of experimental plots is less than the number of plots set in a standard experimental layout. SHP Buddy provides a streamlined method for shapefile generation in QGIS by arranging plots in a serpentine pattern and automatically removes fill plots at the end of replicates and in irrigation wheel tracks. Irrigation system wheel tracks occur when the wheels of lateral irrigation systems go through plots in an experiment. Researchers typically anticipate this by including fill plots in this range within the experiment. Additionally, plot sizes are adjustable to exclude unwanted features such as alleyways and vegetation from adjacent plots. SHP Buddy can incorporate plot information directly from field book CSV files that are typical in breeding programs without the need for a field map CSV intermediate. Lastly, SHP Buddy is a free and open-source software that can be installed directly from the QGIS plugins repository from within the program thereby lowering the barrier to entry and reducing the possibility of installation errors.

## Implementation

### Installation

SHP Buddy can be directly installed within QGIS from the QGIS Python Plugins Repository. The plugins repository can be accessed through selecting the ‘Plugins’ dropdown and then ‘Manage and Install Plugins’. The plugin may be found by typing “SHP Buddy” in the search box on the ‘All’ tab to install.

### Architecture

SHP Buddy was initially tested and debugged in QGIS (v3.38.3, QGIS Development Team) using imagery and experiment field layouts from the University of Georgia Soybean Breeding and Genetics program. The initial template files were generated using ‘QGIS Plugin Builder’ (v3.2.1, GeoApt, LLC.). The graphical user interface was designed in Qt Designer (v.5.15.13, The Qt Company, Ltd.). The plugin code was written in Python (v3.9, Python Software Foundation) using the PyCharm Community Edition (v2022.1.2, JetBrains) integrated development environment. The Python QGIS API was used to access processing functions for the geospatial data. Some sections of the Python code were written with the assistance of ChatGPT (vGPT-4, OpenAI).

### Plot numbering

SHP Buddy generates field plot layouts in a serpentine pattern beginning at the bottom left of the shapefile and increasing across each range before moving to the next range. The directionality of plot numbers (i.e. right to left or left to right) can be switched within the interface. Replicated experiments are numbered such that the last two or three digits indicate the plot number within each replicate for experiments with less than 100 or 100 or more plots per replicate, respectively. The leading plot number digits indicate the replicate number. For example, a three-replicate experiment with 20 plots each is numbered 101–120, 201–220, and 301–320. Similarly, a three-replicate experiment with 200 plots each is numbered 1001–1200, 2001–2200, and 3001–3200. SHP Buddy assumes that replicates are balanced. Unreplicated experiments are numbered sequentially from a first and last plot number indicated by the user. The plot numbers can be any number of digits.

## Discussion

The SHP Buddy program provides an intuitive and convenient way to quickly generate shapefiles for common experiment layouts used in agricultural research programs. The main advantage of this plugin compared to other comparable shapefile generation solutions is the ease of installation. Other solutions require some degree of programming knowledge for proper installation, such as the use of console commands, Python, and Git. In contrast, SHP Buddy is available on the QGIS plugin repository and can be installed with only a few clicks within the QGIS interface. Further, SHP Buddy does not have any Python package dependencies that need to be installed outside of QGIS, mitigating the risk of compatibility errors. These qualities result in SHP Buddy having a substantially lower learning curve than other options.

Further, to our knowledge this is the first published program of its type to allow for the incorporation of field book information into shapefiles without the need for an intermediate field map file indicating the positions of plot numbers. Instead, SHP Buddy incorporates this information directly from a field book CSV file. This further reduces the amount of time needed for shapefile generation by eliminating the need for supplementary files especially for agricultural research programs with a large number of experiments.

### Interface

**Fig. 1:**
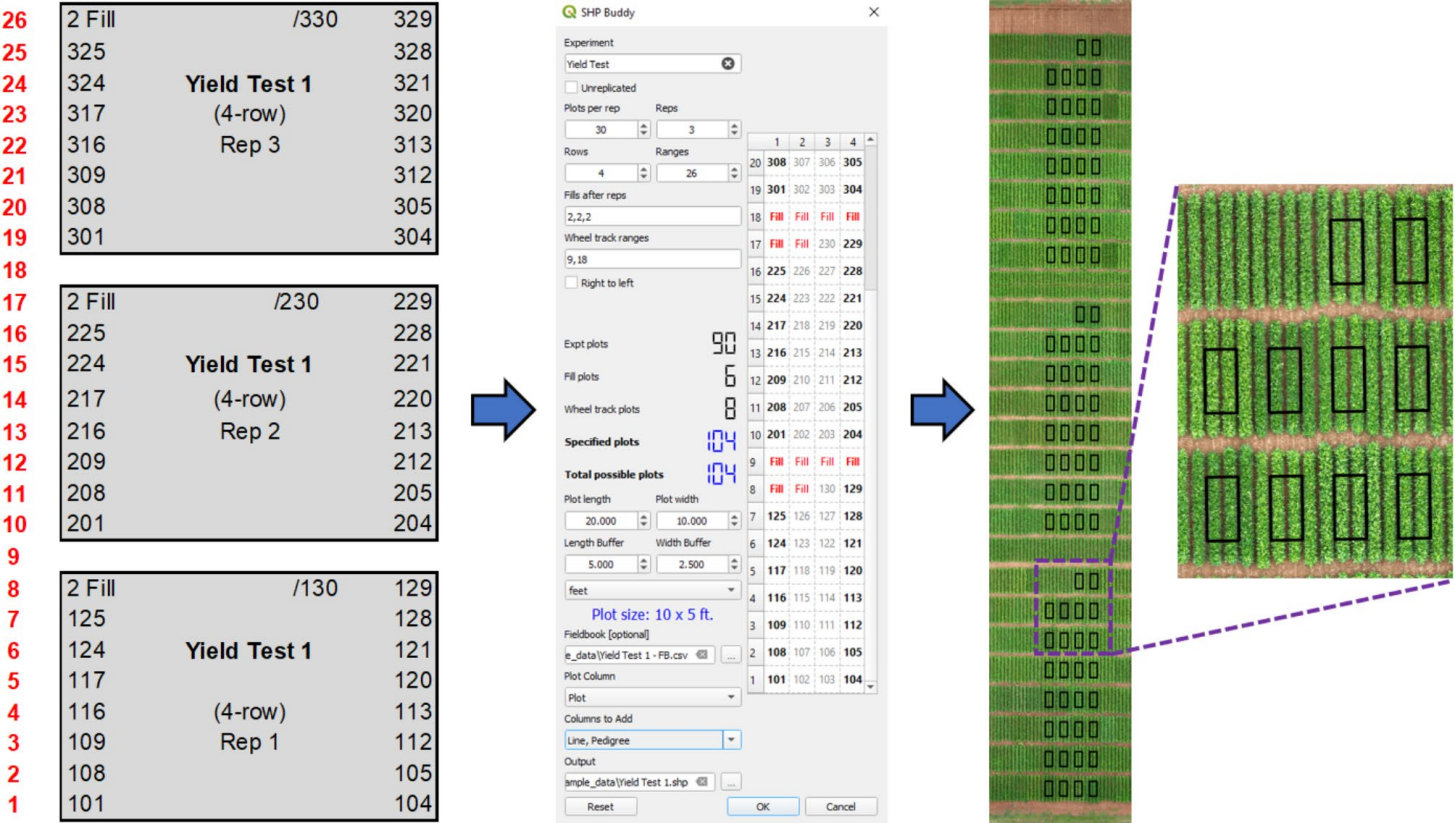
Workflow for generating shapefile for Yield Test 1. The experiment layout specifications illustrated in the field map (left) are input into the SHP Buddy dialog (center), including adding a field book CSV file with desired headers selected. The resulting shapefile can be aligned with the aerial field image (right)

**Fig. 2:**
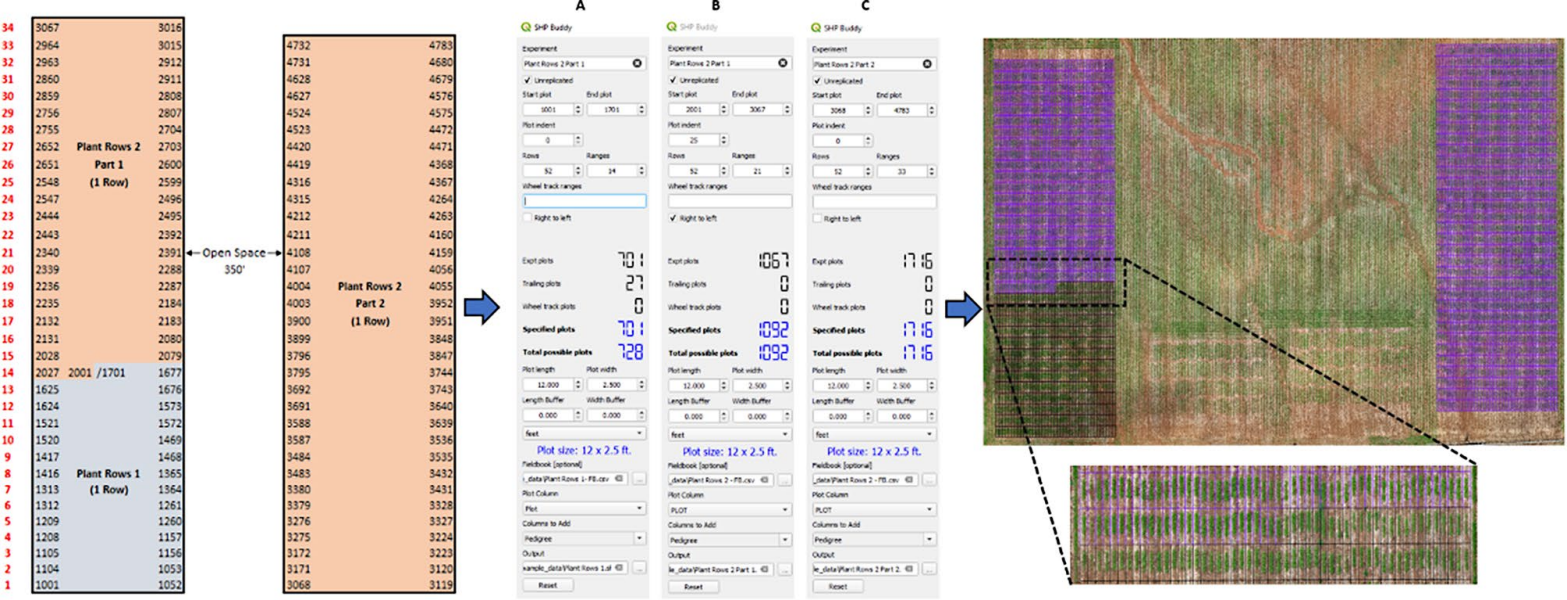
Workflow for generating Plant Rows set #1 (black, bottom) and set #2 (purple, bottom) nurseries. The nursery layout specifications illustrated in the field map (top left) are input into the SHP Buddy dialog (Plant Rows set #1: **A**; Plant Rows set #2 Part 1: **B**; Plant Rows set #2 Part 2: **C**), including adding a field book CSVs with desired headers selected. The resulting shapefiles can be aligned with the aerial field image (bottom)

The plugin can be opened by clicking the plugin icon on the plugin toolbar or from the ‘Vector’ dropdown on the toolbar. The plugin interface is shown in Figs. 1 and 2 associated with the case studies described in the subsequent section. The experiment name can be optionally specified in the first field. By default, the window will display options for designing a replicated experimental layout. Options for unreplicated experiments are displayed when the corresponding checkbox is toggled.

Replicated designs take six numeric inputs. ‘Plots per rep’ indicates the number of plots in each replicate. SHP Buddy assumes that each replicate consists of the same number of experimental plots. The total number of replicates is indicated in the “Rep” box. The “Rows” and “Ranges” values indicate the total dimensions of the experiment in terms of the number of plots in each range and total number of ranges, respectively. The range value should account for skipped ranges (for example, due to irrigation wheel tracks) between ranges within an experiment. “Fills after reps” is a list of numbers indicating the number of non-experimental, or fill, plots after each replicate. The length of the list must equal the value indicated in “Reps” even for experiments without fill plots. “Wheel track ranges” is an optional numeric list indicating the ranges within an experiment that are skipped. For example, a value of “9,18” indicates that the 9th and 18th ranges from the front of the experiment are skipped. Each number in this list must be less than or equal to the value indicated in “Ranges”. Lastly, the “Right to left” check box inverts the plot order from the default of increasing from left to right. The number of user-specified plots can be tracked on the LCD panel below the inputs. For replicated experiments, the “Specified plots” value must match the “Total possible plots”, the former being the sum of experimental, fill, and wheel track plots, while the latter being the product of the rows and ranges values. Warnings will be displayed to indicate issues with the input values. A preview of the map will be displayed if there are no errors. This map represents a matrix generated internally by the code. SHP Buddy automatically removes plots designated as fill plots when the shapefile is generated.

Plots in unreplicated layouts will be numbered sequentially from the “Start plot” to “End plot” value. “Plot indent” is an optional value indicating the number of skipped plots in the first range preceding the first plot. This scenario can occur when a preceding experiment does not fill the last range. The LCD layout is slightly modified for unreplicated experiments to indicate the number of excluded plots in the final range. The “Specified plots” value must be less than the “Total possible plots” value. Other checks have been added to prevent errors during shapefile generation. A map preview will appear if no errors are detected.

The next section of the window is for designating plot dimensions and buffers. “Plot length” and “Plot width” indicates the total dimensions of each plot, including all rows and alleyways. The final plot size can be trimmed with the “Length buffer” and “Width buffer” values. The total dimension is reduced by twice its respective buffer. The user can specify the English or metric system as the units of the dimensions and buffers. Lastly, the final plot dimensions expressed as length by width will be displayed below the dimension inputs.

Below, a field book CSV file can be optionally indicated. Field book CSVs must have a first row of column headers with one column containing all of the plot numbers displayed in the field map preview. This column is indicated by the “Plot Column” dropdown. Next, the user can select any additional columns from the field book to add to the shapefile in the “Columns to Add” dropdown. Lastly, the “Output” field optionally designates a pathway and file name for the resulting shapefile. The shapefile will be added as a temporary layer if this field is left blank. Pressing “OK” will generate the shapefile.

The shapefile is added as a layer at the center of the QGIS canvas and can be further manipulated using native QGIS tools. The location of the shapefile can be adjusted by selecting the shapefile layer and pressing the “Toggle Editing” button on the digitizing toolbar. Individual plots can be adjusted by pressing the “Move Feature” or “Rotate Feature” tools followed clicking the desired plot. The entire shapefile can be adjusted by selecting all plots with the “Select All Features” tools in the selection toolbar and then using the aforementioned adjustment tools. Modifying the entire shapefile is useful for aligning the shapefile with the plots in the underlying raster whereas modifying individual plots is useful for oddities within the field image such as shifted plots resulting from planting errors or lack of georeferencing.

SHP Buddy was designed to be compatible with the most common experiment layouts used in plant breeding or other agricultural research experiments. Possible scenarios are described in the following sections. Example field maps, field book CSVs, and shapefiles can be accessed from the *example_data* folder in the SHP Buddy GitHub repository (https://github.com/nburner96/shp_buddy).

### Case study 1: replicated field trial

The first example is of a typical replicated experiment that might be common during the yield testing stage of a breeding pipeline (Fig. 1). This experiment consists of three replicates, each consisting of 30 plots. Each replicate is trailed by two fill plots and irrigation wheel tracks are present in ranges 9 and 18. The total footprint of this experiment is 4 by 26 plots. Plots consist of four 2.5 ft rows on a bed size of 20 ft, for a total plot size of 20 × 10 ft. However, in this example the plot width and length will be decreased 2.5 and 5 ft. on each side to more precisely capture the canopy of each plot. The final plot size will therefore be 10 × 5 ft. The line name and pedigree information for each plot will be added from the field book CSV file. The experiment map, SHP Buddy settings, and resulting shapefile are shown in Fig. 1.

### Case study 2: unreplicated trials

The second example is of two unreplicated trials based on two early generation nurseries (Fig. 2). The layouts of unreplicated trials such as breeding nurseries tend to be more ad hoc than those of replicated trials due to less concern to the field effects due to a reduced selection intensity or serving as generation advancement or seed increases. In this example, Plant Rows set #1 consists of 701 single row plots, spaced 2.5 ft apart planted on 12 ft. beds. Plant Rows set #2 begins after the last plot of Plant Rows set #1, and as such starts 25 plots into the range and continues for 1,067 plots. However, because Plant Rows set #1 ends in the middle of a range, Plant Rows set #2 Part 1 begins with plot numbers increasing from right to left. Due to a washed-out area of the field, Plant Rows set #2 Part 2 continues for 1,716 plots 350 ft. to the right of the aforementioned sections. Each section is 52 plots wide and 34 and 33 ranges long for the first and second sections, respectively. Despite being broken into two parts, the same Plant Rows set #2 field book can be used for both parts. It is worth noting that the entirety of Plant Rows set #2 could be made in one run by using the total number of ranges and adjusting the end plot number. The result could then be manually split and moved to the appropriate field locations.

### Advancement of features

Two comparable programs, BreederMap and FIELDimageR-QGIS that work directly in QGIS for shapefile generation for agricultural experiments have been reported [17, 19]. Compared to these programs, SHP Buddy provides key advantages that enhance shapefile generation for plant breeding experiments. It provides automated shapefile precision without manual scaling by determining the experiment extents based on the number of plots and their dimensions, eliminating the need for manual scaling during shapefile generation. Besides, SHP Buddy simplifies data integration with an intuitive plot numbering system that is easily incorporated into field book CSVs. This approach reduces complexity and minimizes errors compared to methods that require separate CSV field map files with matrix configurations. Also, SHP Buddy automates plot layout calculations, preventing errors in coordinate collection or marking corners. This is particularly advantageous for row-crop experiments, where maintaining a precise rectangular grid is critical. Lastly, SHP Buddy’s methodology is designed for fields with numerous experiments, offering a scalable solution for multiple experiments that eliminates unnecessary manual steps, saving time and effort for researchers to manage complex field layouts. Overall, SHP Buddy offers a simpler, and more intuitive and precise method to generate shapefiles.

### Limitations

SHP Buddy is designed to accelerate the process of shapefile generation for common serpentine plant breeding experiments. However, there are some features that may be of interest to other researchers that are not currently implemented. First, SHP Buddy is designed for experiments with balanced replicates. For unbalanced experiments, it may be advised to use the unreplicated methodology for each individual replication. Otherwise, one could create a replicated shapefile where the number of plots in each replicate is equal to the maximum number of plots across replicates. The user could delete unnecessary plots and move and adjust the remaining plots as necessary.

An additional possible limitation is the use of custom plot IDs. It is recommended that a custom plot ID column is added to the field book CSV that matches custom plot IDs to the prescribed SHP Buddy plot numbering system. We believe that the SHP Buddy numbering system is intuitive and is based on typical experiment layouts. Therefore, it should not cause too much difficulty to match custom plot IDs to the SHP Buddy plot IDs in the field book CSVs.

Lastly, SHP Buddy lays out plot numbers in a serpentine pattern across ranges. However, some experiments may choose to serpentine across rows. That is, one would walk parallel to the rows when taking notes of sequential plots as opposed to parallel to ranges. This type of layout can be generated with SHP Buddy, but would require the shapefile to be rotated 90 degrees after it is generated. Additionally, the plot length and width specifications would have to be swapped to account for this transformation.

## Conclusions

Shapefiles are necessary components of any high throughput phenotyping pipeline for demarcating regions of interest in an image. SHP Buddy vastly reduces the time that it takes to extract spectral data from aerial imagery by providing a quick and accurate way to generate field plot shapefiles. SHP Buddy improves upon comparable programs by providing an intuitive user interface for generating precise shapefiles and incorporating field book information. A repeatable method for shapefile generation helps avoid mislabeling plots which is an error that can often go unnoticed during manual shapefile creation. More accurate shapefiles will improve the quality of high-throughput phenotyping data used by researchers. Further, the shapefiles generated by SHP Buddy can serve as a useful record of previous fields by allowing for easy identification of field plots of interest by researchers. Time-saving methodologies such as those provided by SHP Buddy makes plant breeding and agricultural research experiments more efficient by allowing more time and resources to be focused on other research activities.

## Data Availability

Example field maps, field book CSVs, and shapefiles can be accessed from the example_data folder in the SHP Buddy GitHub repository (https://github.com/nburner96/shp_buddy).

## Availability and requirements

Project Name: SHP Buddy.

Project home page: https://github.com/nburner96/shp_buddy.

Operating system: Windows.

Programming language: Python.

Other requirements: QGIS 3.0 or higher.

License: MIT.

SHP Buddy is a free and open source QGIS plugin. SHP Buddy may be used, modified, and distributed with proper acknowledgement of the original publication and authors. Research that benefitted from SHP Buddy’s functionality should cite the original publication.

## Abbreviations

QGIS: Quantum geographic information system
SHP: “Shape”, as in.shp file
UAV: Unmanned aerial vehicle
